# Supplementary material for: On the robustness of the emergent spatiotemporal dynamics in biophysically realistic and phenomenological whole-brain models at multiple network resolutions
Source: Front Netw Physiol. 2025 Aug 8;5:1589566. doi: 10.3389/fnetp.2025.1589566 (PMC12371574; doi:10.3389/fnetp.2025.1589566)
Supplement: Supplementary file 1 [file DataSheet1.pdf]

Supplementary Material

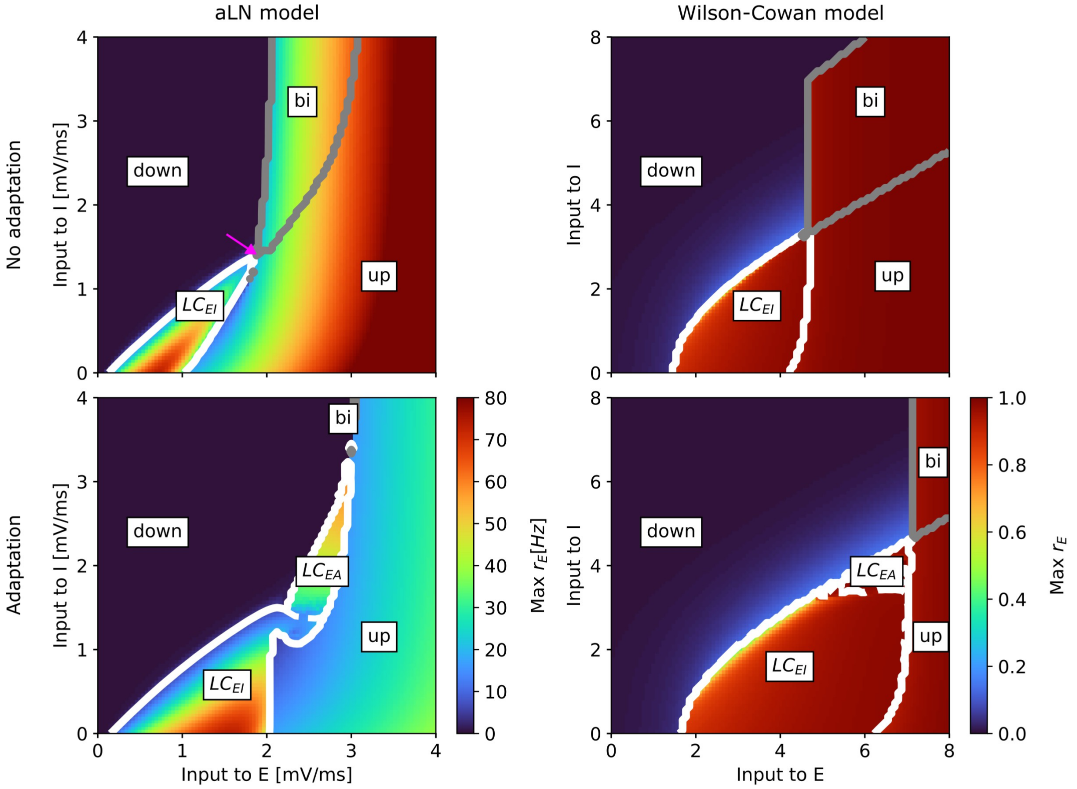

Figure A1: Slice of state space of the single-node aLN (left column) and Wilson-Cowan (right column) models without (b = 0; top row) and with adaptation (b = 20 pA, 60; bottom row) spanned by the external input currents to the E and I populations. In every panel, the horizontal (vertical) axis denotes the external input current μ_E^{ext} (μ_I^{ext}) to the excitatory (inhibitory) population. The heatmap shows the maximum excitatory firing rate r_E of the model. State boundaries are indicated by solid white lines for the fast (LC_{EI}) and slow (LC_{EA}) oscillatory regions, and by solid grey lines for the regime of bistability between up and down states (*bi*). The magenta arrow indicates the region where few points with bistability between the up and the fast (LC_{EI}) state are found. Unistable up (up) and down state (down) regions are also marked. Model parameters are given in Tables 1 and 2.

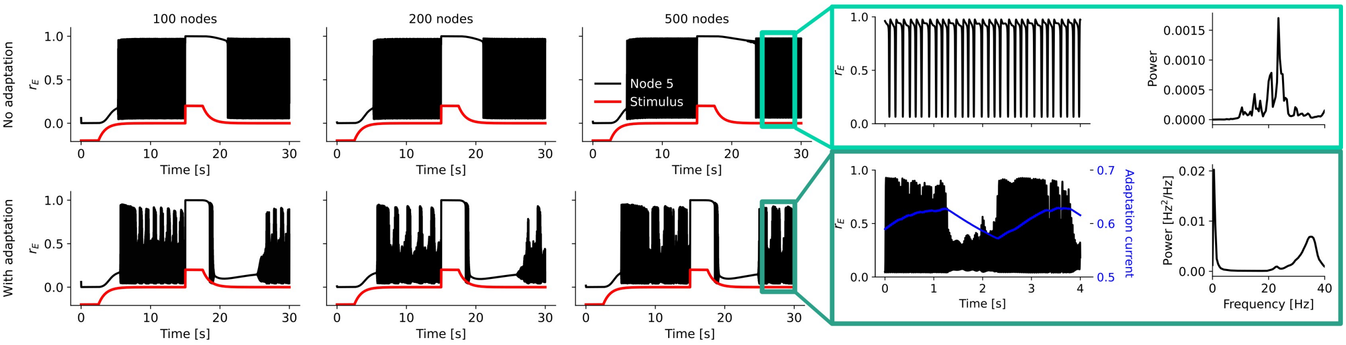

Figure A2: Example time series of the proportion r_E of active neurons per unit of time of one randomly chosen node (black line) of the whole-brain Wilson-Cowan model at several points in state space, without ($b = 0$; top row) and with ($b = 60$; bottom row) adaptation for a network with 100 (left column), 200 (middle column), and 500 nodes (right column). Parameters are as follows: 100 nodes - $\mu_E^{ext} = 4.3$, $\mu_I^{ext} = 2.8$; 200 nodes - $\mu_E^{ext} = 4.3$, $\mu_I^{ext} = 2.85$; 500 nodes - $\mu_E^{ext} = 4.2$, $\mu_I^{ext} = 2.7$. The light (top) and dark green (bottom) insets display enlarged intervals of the time series of the proportion r_E of active neurons per unit of time and, in case of finite adaptation, the current I_A for the chosen node, and also show the power spectrum for the brain network with 500 nodes averaged across all nodes. All other model parameter values are given in Table 2.

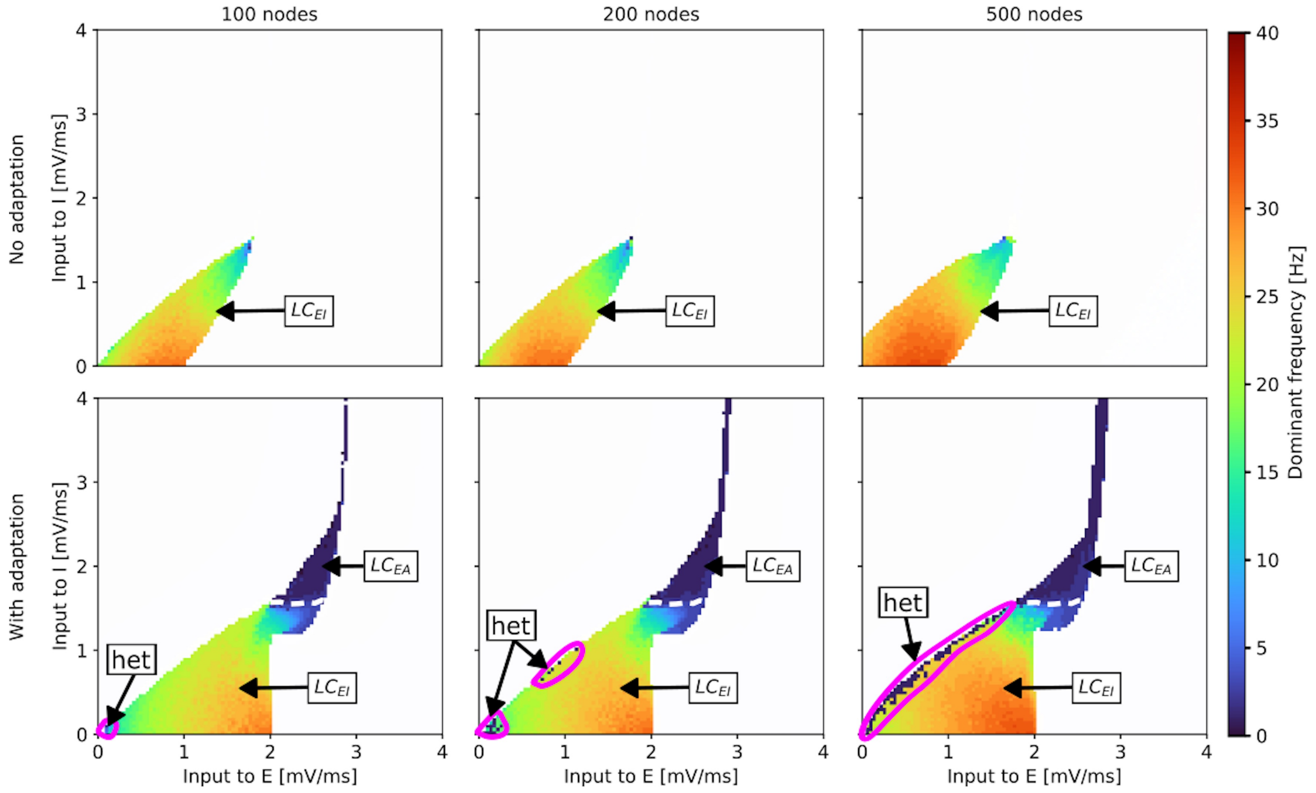

Figure A3: Frequency of the peak of the power spectrum averaged over all nodes of the whole-brain aLN model without (b = 0 pA; top row) and with (b = 20 pA; bottom row) adaptation for a brain network with 100 (left column), 200 (middle column), and 500 (right column) nodes as a function of the external input current to the E and I populations. In every panel, the horizontal axis shows the external input current to the excitatory population (μ_E^{ext}) and the vertical axis shows the external input current to the inhibitory population (μ_I^{ext}). The average dominant frequency (Hz) across all nodes in the network is color-coded. The white dashed line indicates the approximate border between the fast LC_{EI} and slow LC_{EA} oscillating regions. The magenta solid lines indicate the areas where heterogeneous (het) slow-fast oscillations were identified (for b = 20 pA). All model parameters are summarized in Table 1.

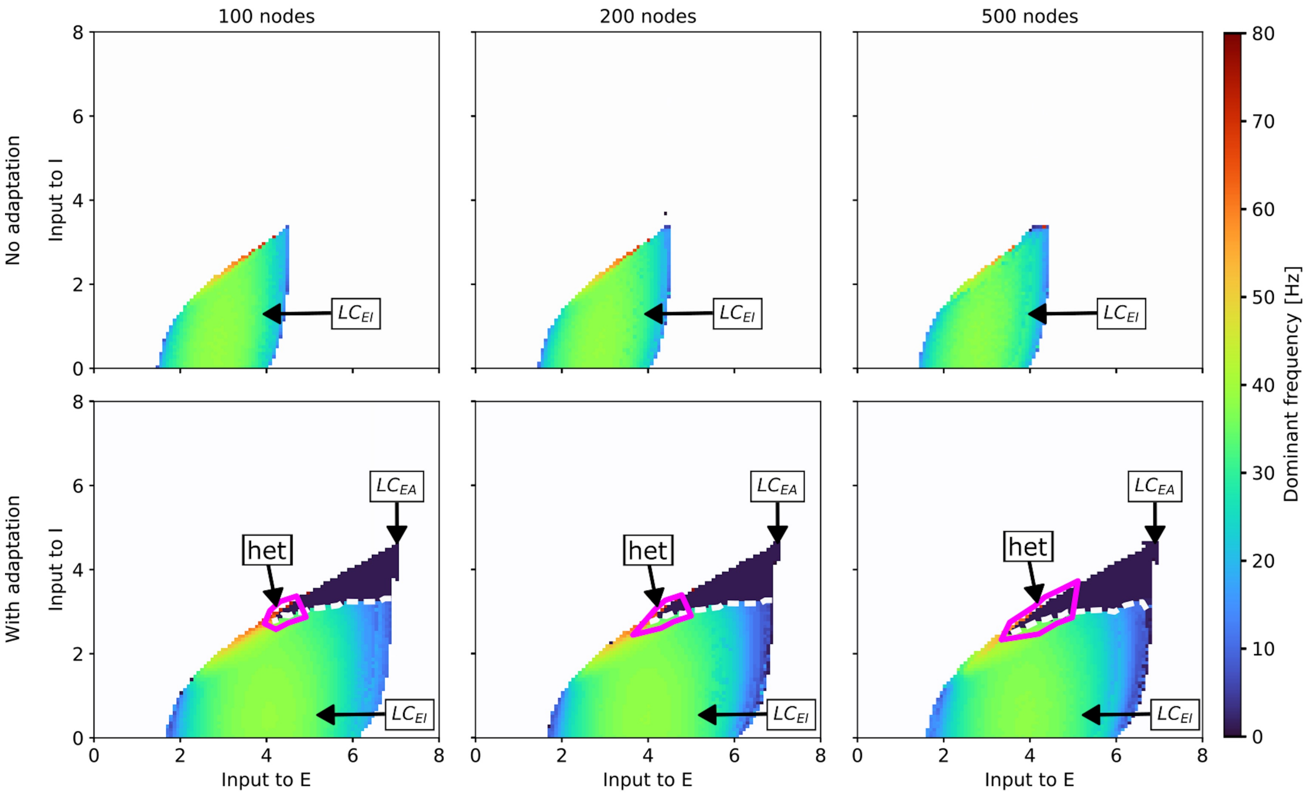

Figure A4: Frequency of the peak of the power spectrum averaged over all nodes of the whole-brain Wilson-Cowan model without (b = 0; top row) and with (b = 60; bottom row) adaptation for a brain network with 100 (left column), 200 (middle column), and 500 (right column) nodes as a function of the external input current to the E and I populations. In every panel, the horizontal axis shows the external input current to the excitatory population (μ_E^{ext}) and the vertical axis shows the external input current to the inhibitory population (μ_I^{ext}). The average dominant frequency (Hz) across all nodes in the network is color-coded. The white dashed line indicates the approximate border between the fast LC_{EI} and slow LC_{EA} oscillating regions. The magenta solid lines indicate the areas where heterogeneous (het) slow-fast oscillations were identified (for b = 60). All model parameters are summarized in Table 2.

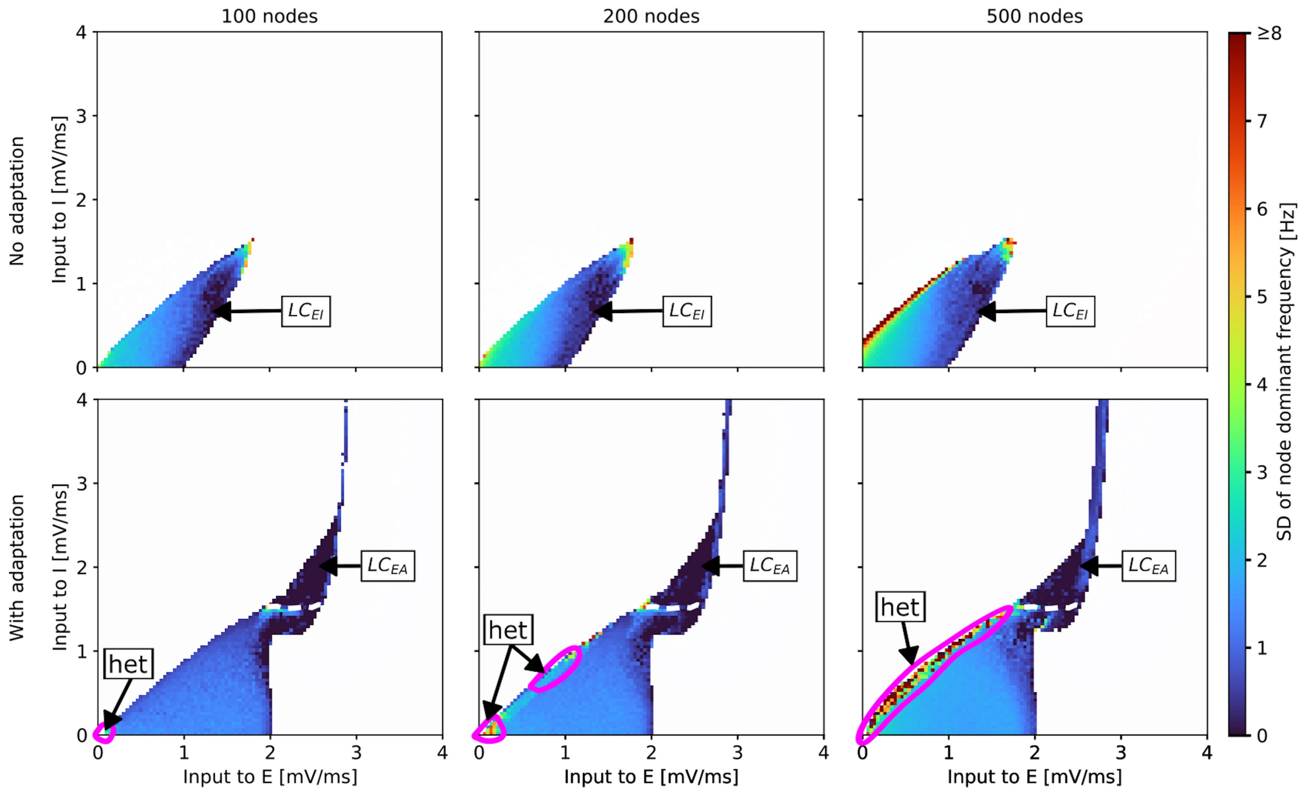

Figure A5: Standard deviation (SD) of the node dominant frequency of the whole-brain aLN model without (b = 0 pA; top row) and with (b = 20 pA; bottom row) adaptation for a brain network with 100 (left column), 200 (middle column), and 500 (right column) nodes as a function of the external input current to the E and I populations. In every panel, the horizontal axis shows the external input current to the excitatory population (μ_E^{ext}) and the vertical axis shows the external input current to the inhibitory population (μ_I^{ext}). The standard deviation of the node dominant frequency (Hz) across all nodes in the network is color-coded. The white dashed line indicates the approximate border between the fast LC_{EI} and slow LC_{EA} oscillating regions. The magenta solid lines indicate the areas where heterogeneous (het) slow-fast oscillations were identified (for b = 20 pA). All model parameters are summarized in Table 1.

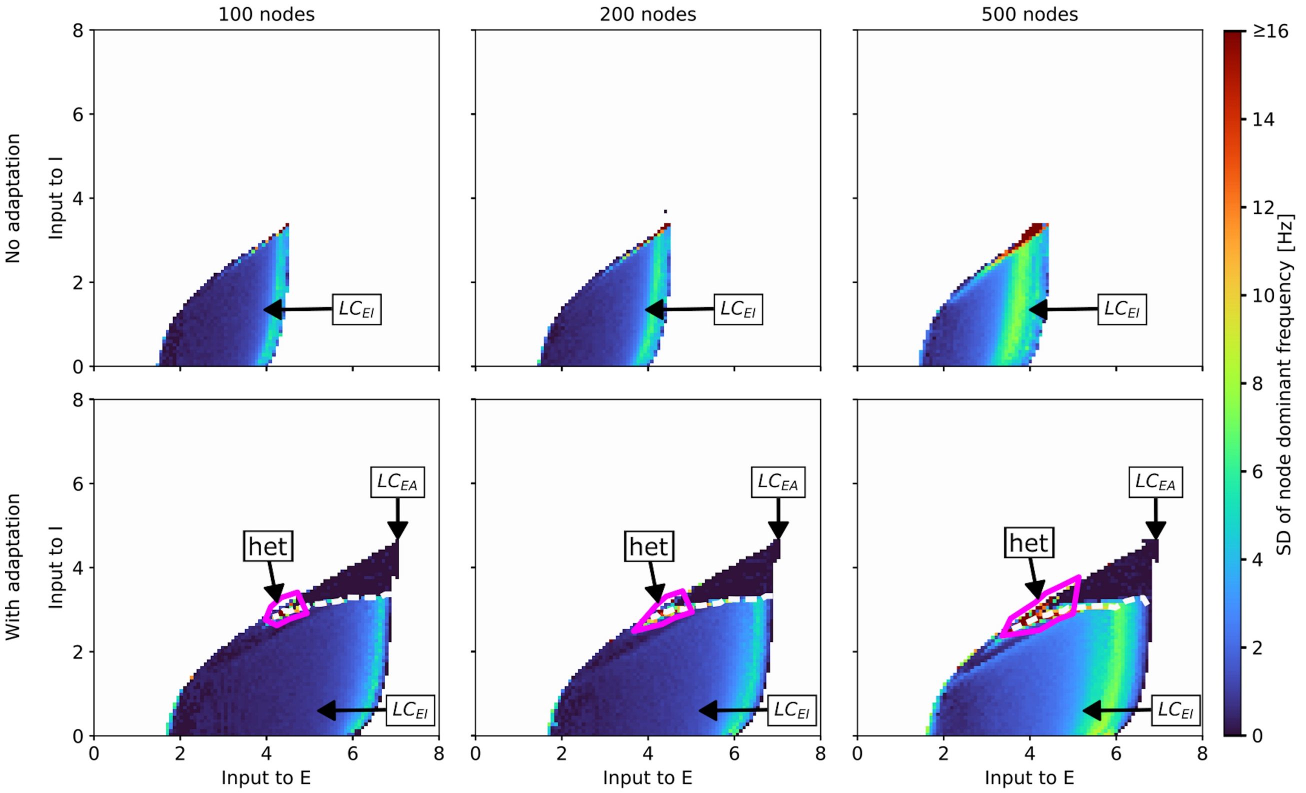

Figure A6: Standard deviation (SD) of the node dominant frequency of the whole-brain Wilson-Cowan model without (b = 0; top row) and with (b = 60; bottom row) adaptation for a brain network with 100 (left column), 200 (middle column), and 500 (right column) nodes as a function of the external input current to the E and I populations. In every panel, the horizontal axis shows the external input current to the excitatory population (μ_E^{ext}) and the vertical axis shows the external input current to the inhibitory population (μ_I^{ext}). The standard deviation of the node dominant frequency (Hz) across all nodes in the network is color-coded. The white dashed line indicates the approximate border between the fast LC_{EI} and slow LC_{EA} oscillating regions. The magenta solid lines indicate the areas where heterogeneous (het) slow-fast oscillations were identified (for b = 60). All model parameters are summarized in Table 2.

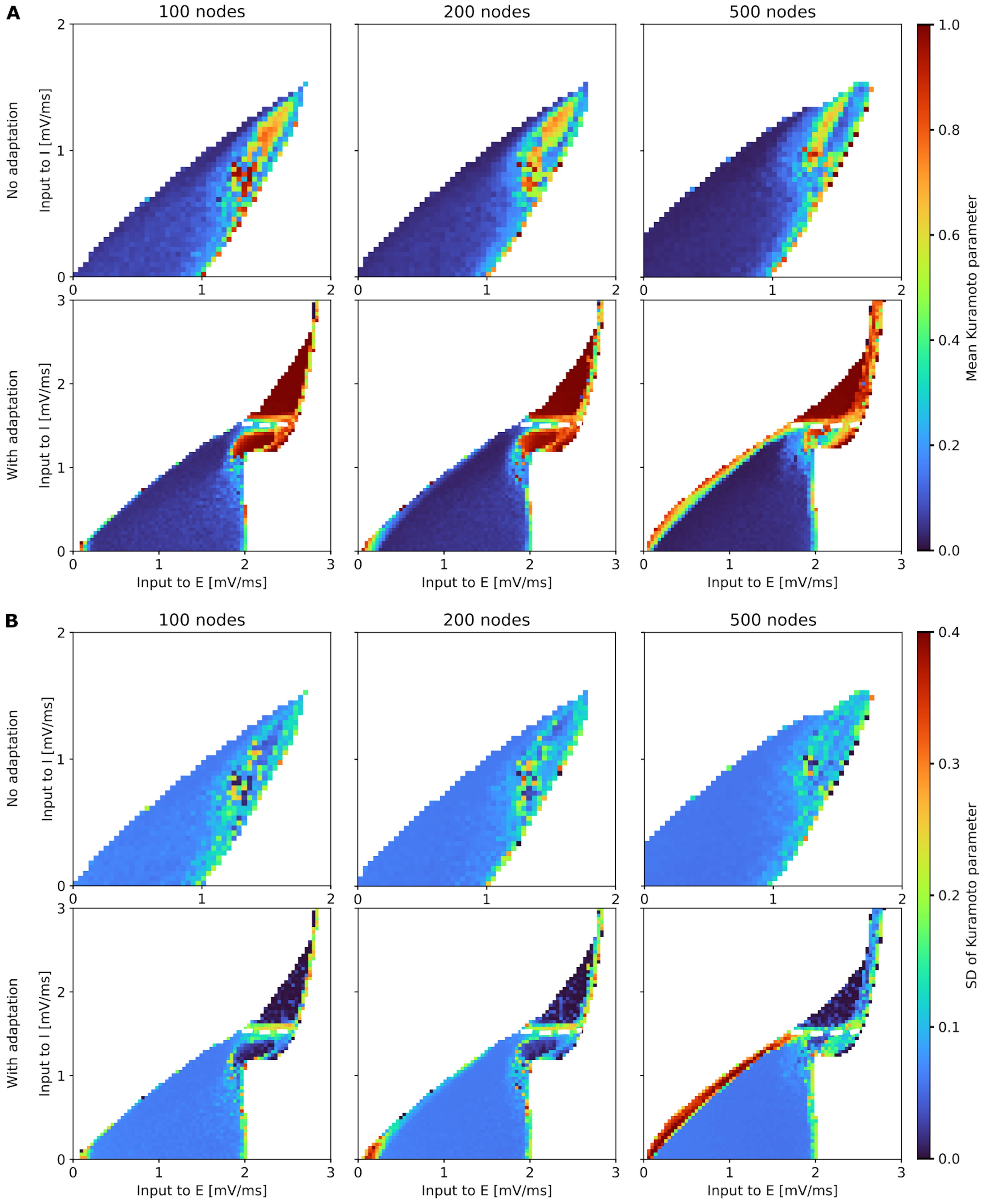

Figure A7: Mean (A) and standard deviation SD (B) of the Kuramoto order parameter for the aLN whole-brain model in the case without (b = 0 pA; top rows) and with (b = 20 pA; bottom rows) adaptation for 100 (left column), 200 (middle column), and 500 (right column) nodes. The slice of state space is spanned by the external input current to the E and I populations. The white dashed lines indicates the approximate border between the fast LC_{EI} and slow LC_{EA} oscillating regions.

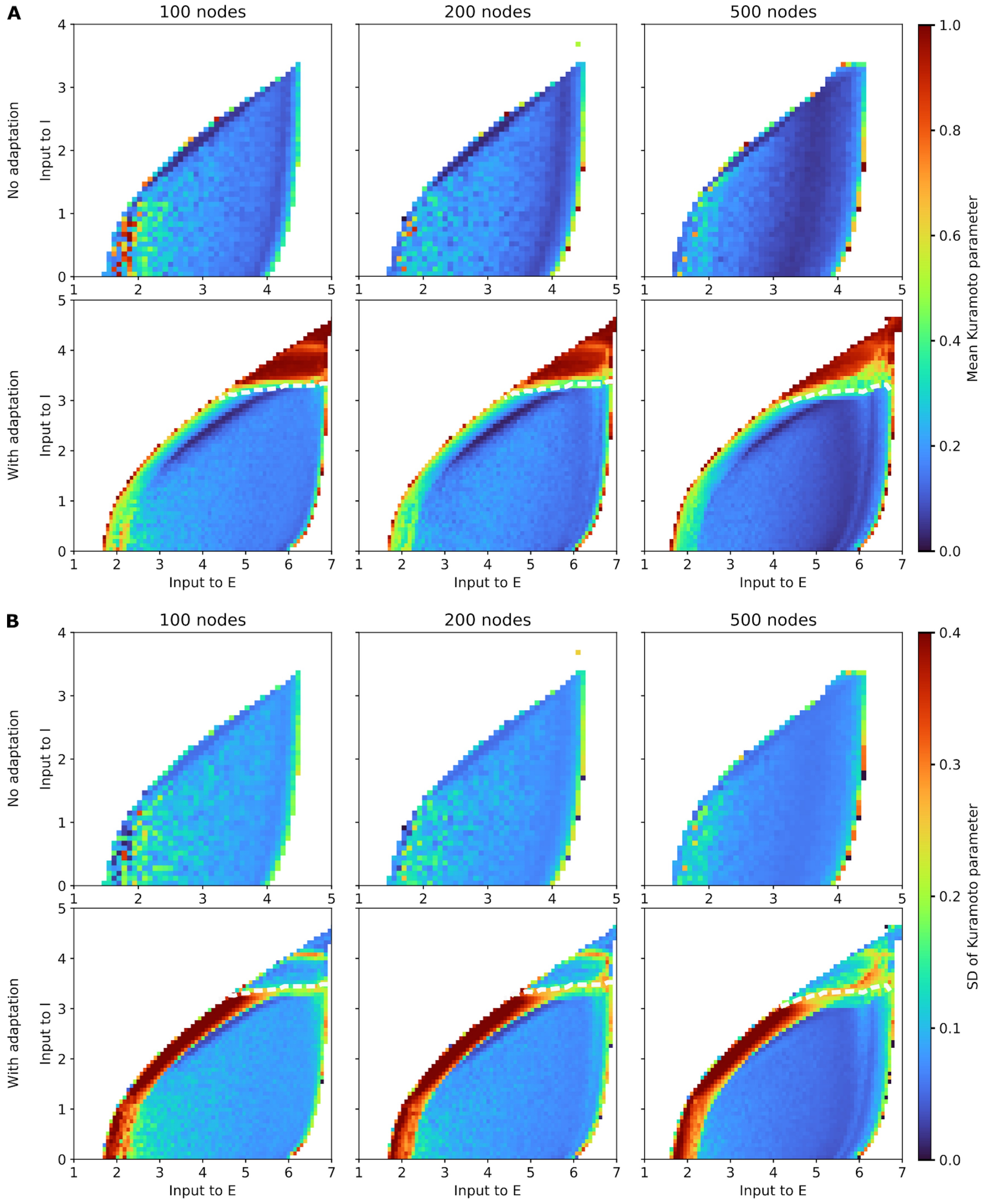

Figure A8: Mean (A) and standard deviation SD (B) of the Kuramoto order parameter for the Wilson-Cowan whole-brain model in the case without (b = 0; top rows) and with (b = 60; bottom rows) spike-triggered adaptation for 100 (left column), 200 (middle column), and 500 (right column) nodes. The slice of state space is spanned by the external input current to the E and I populations. The white dashed lines indicates the approximate border between the fast LC_{EI} and slow LC_{EA} oscillating regions.

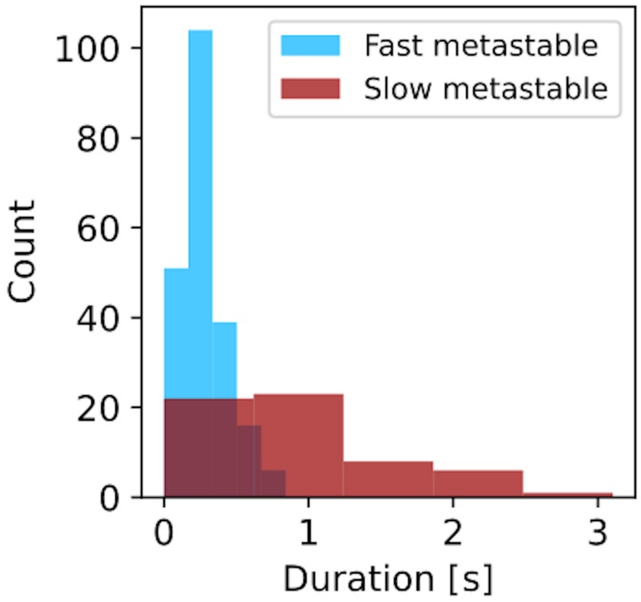

Figure A9: Histogram of the state durations for one slow metastable (red; location C in Figure 6) and one fast metastable point (blue; location B in Figure 6) in state space for the aLN model with 100 nodes and without adaptation ($b = 0$ pA). All other parameters are given in Table 1.

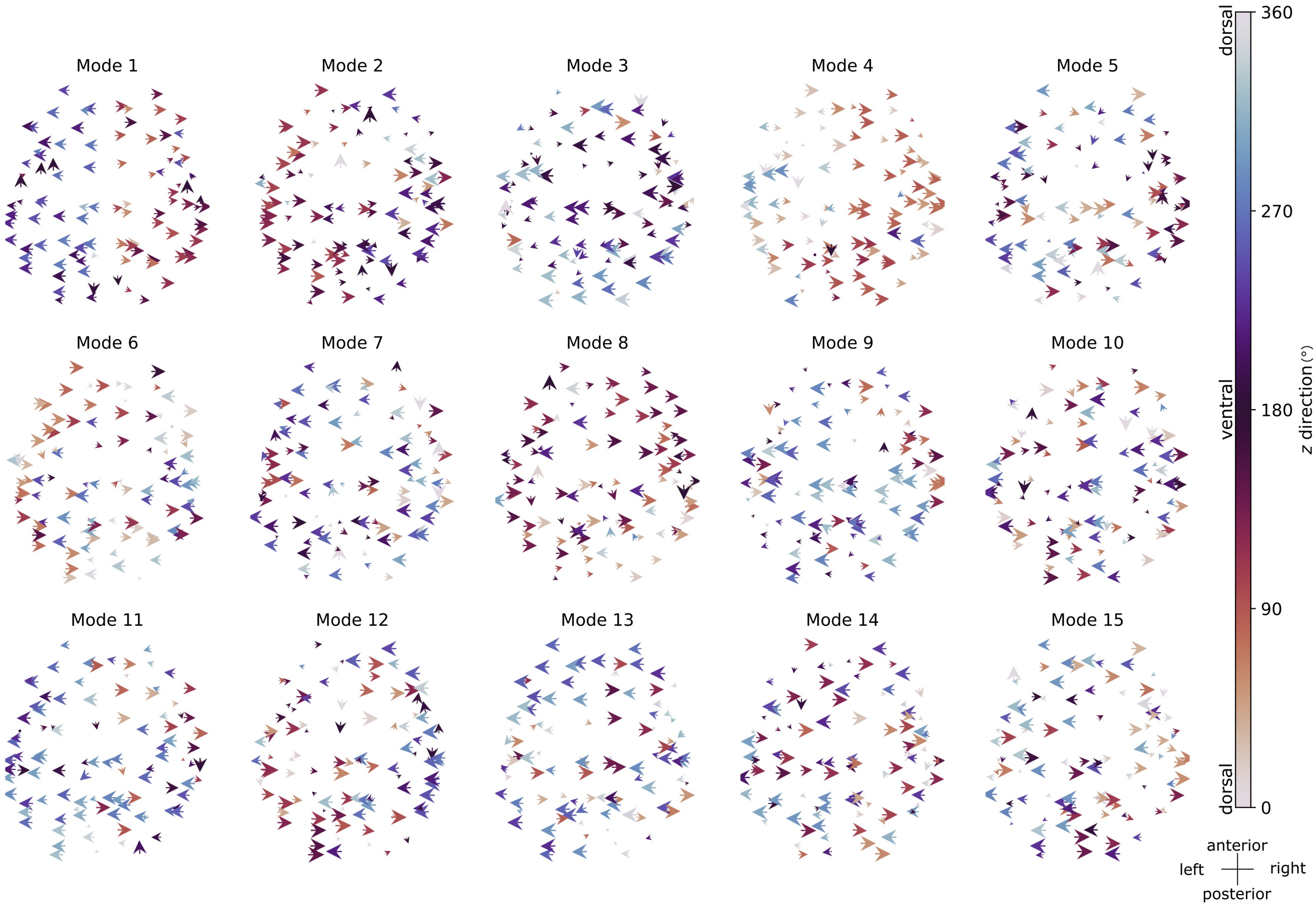

Figure A10: First 15 modes obtained from the singular value decomposition of the velocity vector fields in the whole-brain aLN model with 100 nodes and adaptation (b = 20 pA) for the unistable states in the LC_{EA} region. Modes are ordered in decreasing order of explained variance.

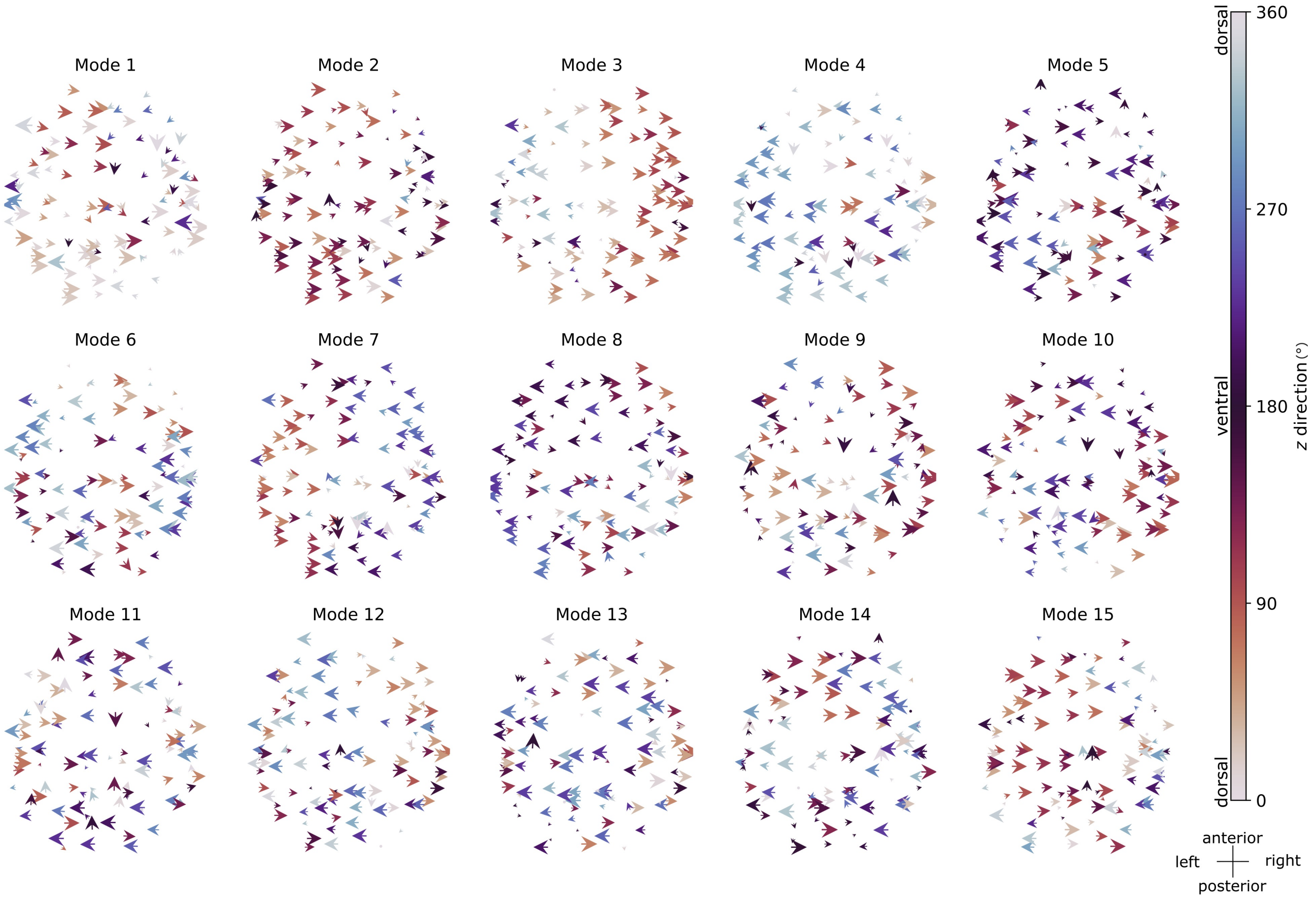

Figure A11: First 15 modes obtained from the singular value decomposition of the velocity vector fields in the whole-brain Wilson-Cowan model with 100 nodes and adaptation (b = 60) for the unistable states in the LC_{EA} region. Modes are ordered in decreasing order of explained variance.

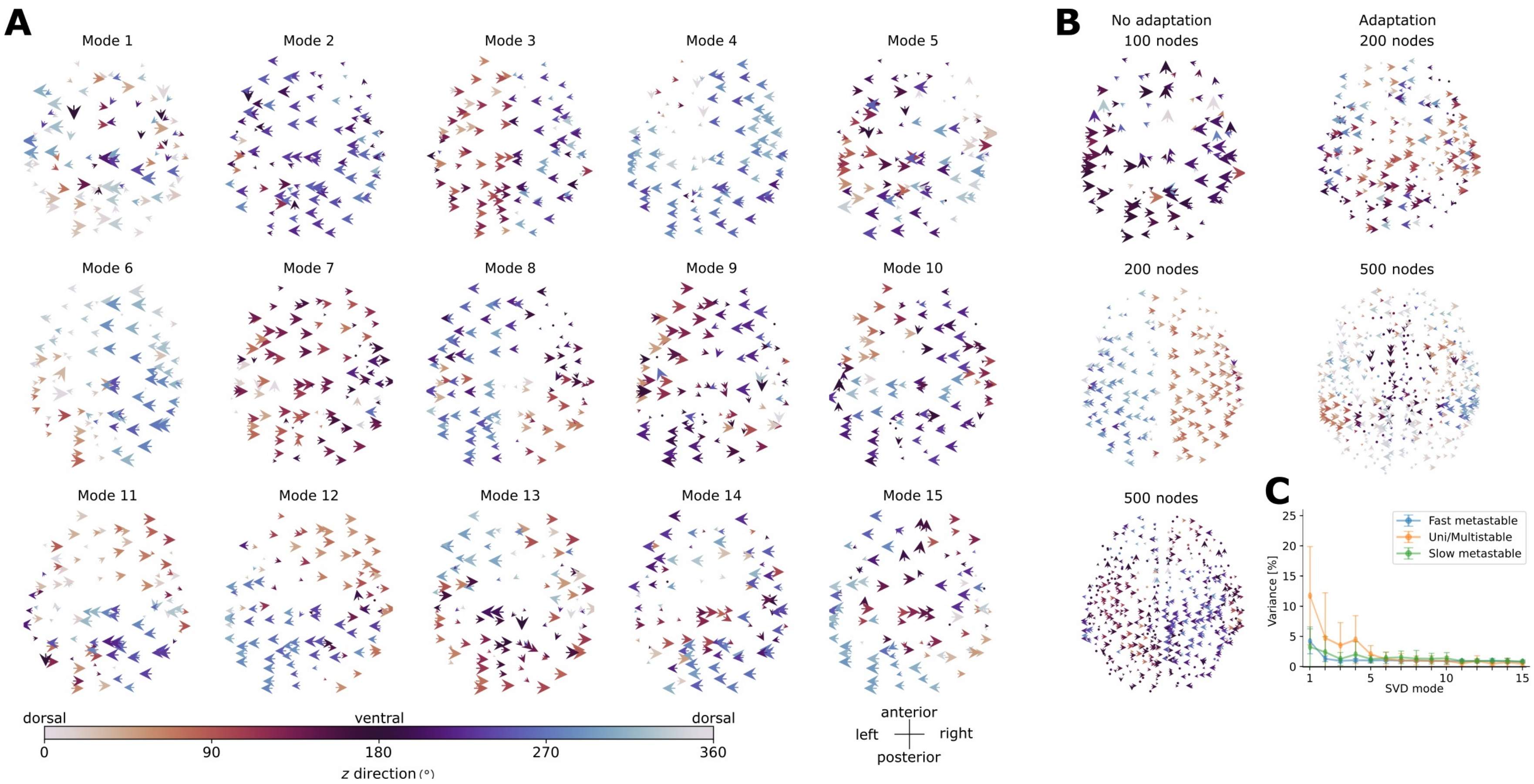

Figure A12: (A) First 15 modes obtained from the singular value decomposition of the velocity vector fields in the whole-brain Wilson-Cowan model with 100 nodes and adaptation (b = 60). Modes are ordered in decreasing order of explained variance. (B) Left panels: modes explaining the largest proportion of variance for the whole-brain Wilson-Cowan model without adaptation (b = 0) with a parcellation of 100, 200, and 500 nodes. Right panels: same as before, but with adaptation (b = 60) and with a parcellation of 200, and 500 nodes. The arrows represent the orientation in the xy plane (left-right and antero-posterior directions) and are color-coded according to the direction along the z-axis (dorso-ventral direction). (C) Percentage of explained variance (mean $\pm$ standard deviation across points in the parameter space) of the first 15 modes identified in (A) for the Wilson-Cowan model with 100 nodes and adaptation (b = 60). The percentage is shown for the different pattern types identified in Section 3.2: uni/multistable (orange), fast metastable (blue), and slow metastable (green).

Table A1. Percentage of explained variance for the dominant 15 modes identified for the aLN model without ($b = 0$ pA) and with ($b = 20$ pA) adaptation for the whole-brain network with 100, 200, and 500 nodes. The column for 100 nodes and $b = 20$ pA corresponds to the modes shown in Figure 9A.

	b = 0 pA			b = 20 pA		
Mode	100 nodes	200 nodes	500 nodes	100 nodes	200 nodes	500 nodes
1	8.42	5.26	5.84	9.31	6.82	3.55
2	6.99	2.10	2.33	7.43	2.76	3.06
3	3.63	1.76	1.70	5.67	1.85	1.63
4	3.31	1.18	1.13	4.74	1.68	1.21
5	2.88	1.04	1.10	3.13	1.26	1.16
6	2.65	1.01	0.83	2.74	1.19	0.99
7	2.20	0.89	0.70	2.57	1.00	0.89
8	1.84	0.76	0.62	1.85	0.88	0.82
9	1.79	0.67	0.53	1.78	0.76	0.70
10	1.58	0.65	0.50	1.63	0.74	0.66
11	1.40	0.61	0.43	1.56	0.69	0.63
12	1.29	0.59	0.40	1.46	0.67	0.62
13	1.19	0.58	0.39	1.32	0.66	0.58
14	1.13	0.55	0.37	1.18	0.64	0.55
15	1.01	0.53	0.37	1.11	0.59	0.49

Table A2. Percentage of explained variance for the dominant 15 modes identified for the Wilson-Cowan model without ($b = 0$) and with ($b = 60$) adaptation for the whole-brain network with 100, 200, and 500 nodes. The column for 100 nodes and $b = 60$ corresponds to the modes shown in Figure A12A.

	b = 0			b = 60		
Mode	100 nodes	200 nodes	500 nodes	100 nodes	200 nodes	500 nodes
1	14.91	13.21	6.64	3.72	11.61	5.14
2	7.60	9.35	5.68	1.58	3.17	0.97
3	3.59	2.86	3.05	1.44	0.97	0.57
4	2.89	2.40	2.32	1.26	0.75	0.49
5	2.31	1.64	1.87	1.19	0.71	0.39
6	1.94	1.49	1.56	1.14	0.65	0.38
7	1.75	1.17	1.44	1.10	0.64	0.34
8	1.59	1.01	1.09	1.06	0.62	0.31
9	1.48	0.91	1.04	1.04	0.55	0.31
10	1.40	0.88	0.92	1.02	0.52	0.30
11	1.15	0.83	0.88	0.95	0.49	0.29
12	1.09	0.76	0.83	0.91	0.47	0.28
13	1.01	0.69	0.71	0.87	0.46	0.27
14	0.98	0.61	0.70	0.84	0.45	0.27
15	0.94	0.59	0.64	0.80	0.45	0.27

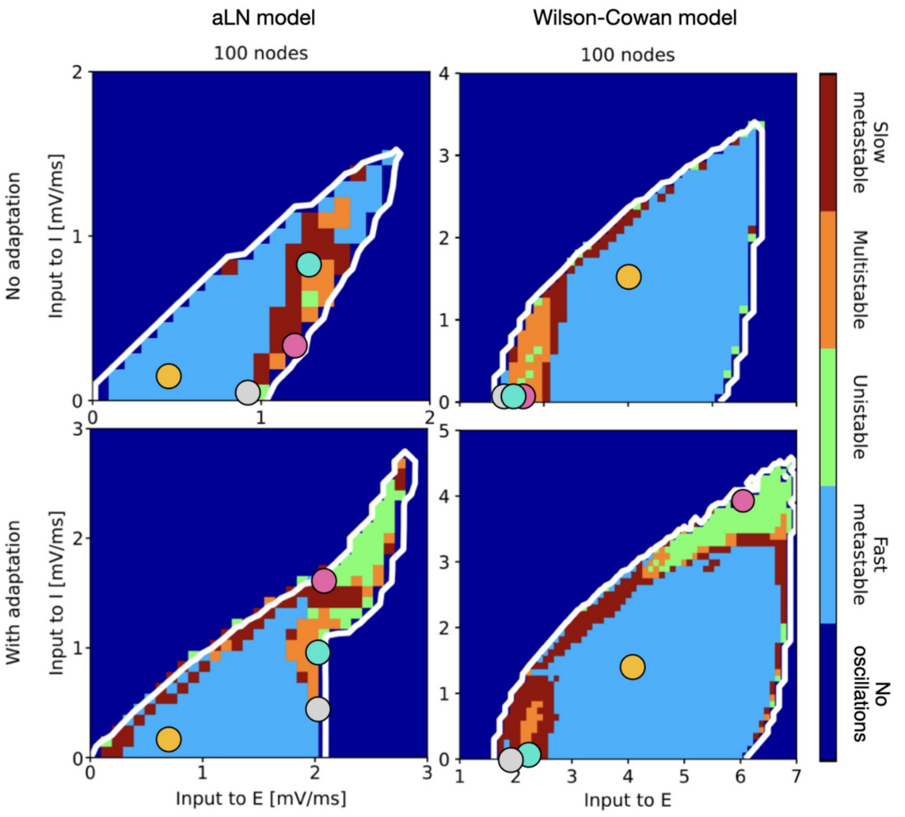

Figure A13: Locations in state space chosen for the results shown in Figures A16 and A17 for the aLN and Wilson-Cowan (wc) models. Multistable state: turquoise, aLN, without adaptation $(\mu_e^{ext}, \mu_i^{ext}) = (1.3, 0.8)$, with adaptation $(\mu_e^{ext}, \mu_i^{ext}) = (2.0, 1.0)$; wc, without adaptation $(\mu_e^{ext}, \mu_i^{ext}) = (1.6, 0.1)$, with adaptation $(\mu_e^{ext}, \mu_i^{ext}) = (2.1, 0.1)$, unistable state: pink, aLN, without adaptation $(\mu_e^{ext}, \mu_i^{ext}) = (1.2, 0.3)$, with adaptation $(\mu_e^{ext}, \mu_i^{ext}) = (2.0, 1.7)$; wc, without adaptation $(\mu_e^{ext}, \mu_i^{ext}) = (1.7, 0.1)$, with adaptation $(\mu_e^{ext}, \mu_i^{ext}) = (6.0, 4.0)$, fast metastable state: yellow, aLN, without adaptation $(\mu_e^{ext}, \mu_i^{ext}) = (0.4, 0.1)$, with adaptation $(\mu_e^{ext}, \mu_i^{ext}) = (0.4, 0.1)$; wc, without adaptation $(\mu_e^{ext}, \mu_i^{ext}) = (3.0, 1.5)$, with adaptation $(\mu_e^{ext}, \mu_i^{ext}) = (4.0, 1.5)$, slow metastable state: grey, aLN, without adaptation $(\mu_e^{ext}, \mu_i^{ext}) = (0.9, 0.0)$, with adaptation $(\mu_e^{ext}, \mu_i^{ext}) = (2.0, 0.4)$; wc, without adaptation $(\mu_e^{ext}, \mu_i^{ext}) = (1.5, 0.0)$, with adaptation $(\mu_e^{ext}, \mu_i^{ext}) = (1.9, 0.0)$. For all simulations and models $b = 0$ in the case of no and $b = 20$ ($b = 60$) for the aLN (wc) model in the case of finite adaptation.

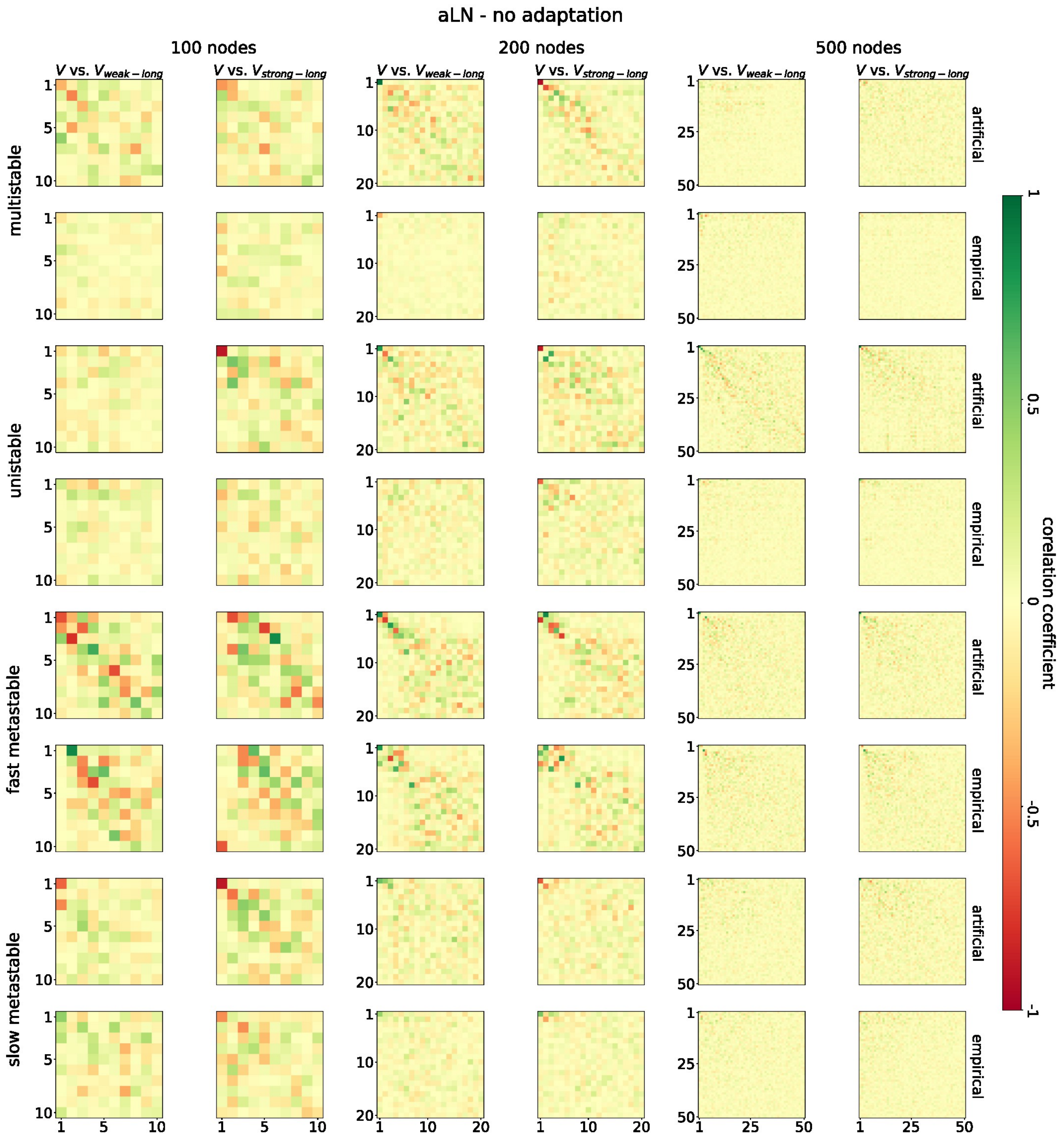

Figure A14: Matrices of correlation coefficients between spatial modes for the aLN model without adaptation. The left (right) column for each parcellation shows the comparison to the case of weak (strong) long-range connections. The upper (lower) rows for each class of states show the comparisons to the case of artificial (empirical) matrices. Colors denote the values of the correlation coefficients.

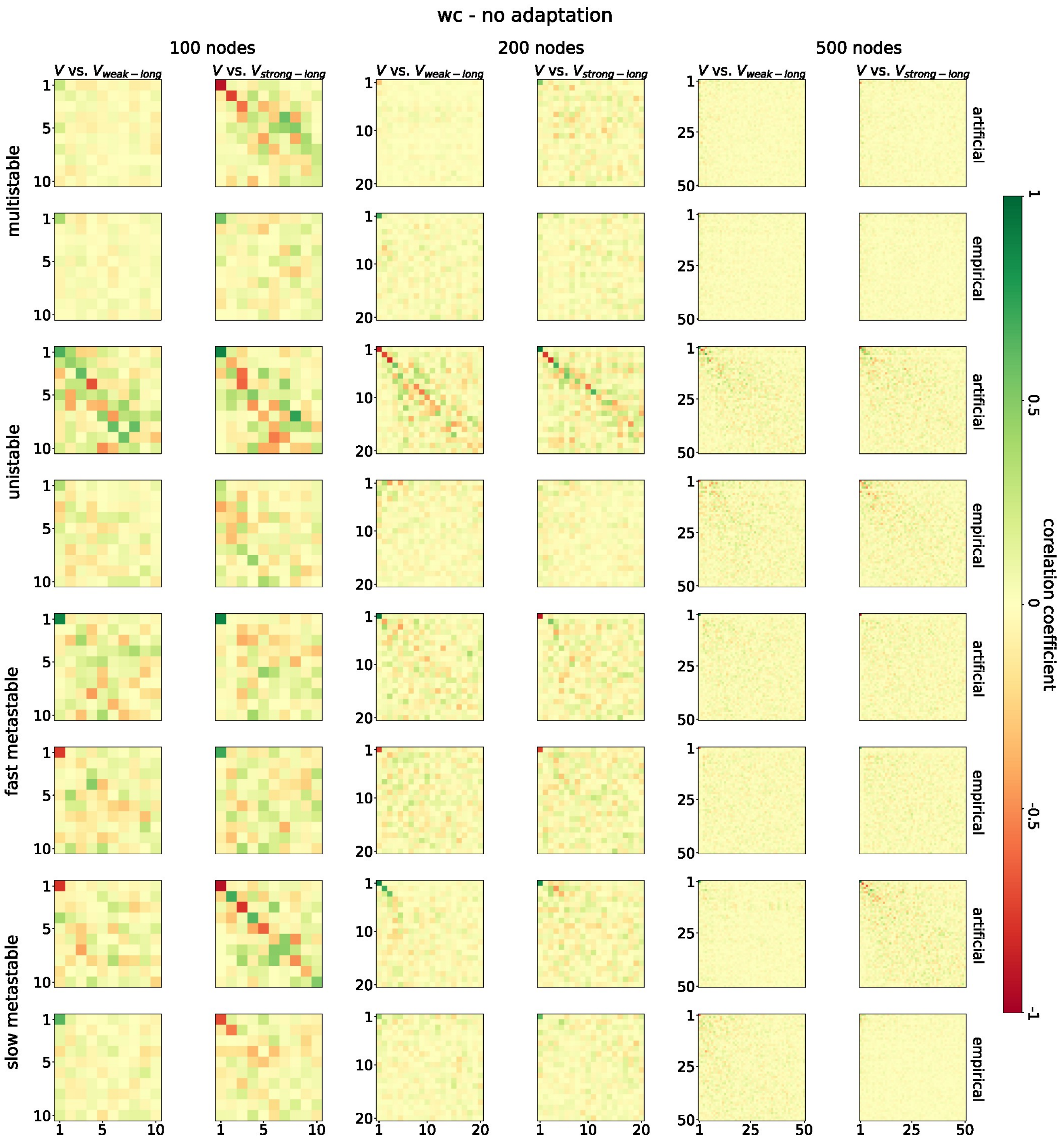

Figure A15: Matrices of correlation coefficients between spatial modes for the Wilson-Cowan model without adaptation. The left (right) column for each parcellation shows the comparison to the case of weak (strong) long-range connections. The upper (lower) rows for each class of states show the comparisons to the case of artificial (empirical) matrices. Colors denote the values of the correlation coefficients.

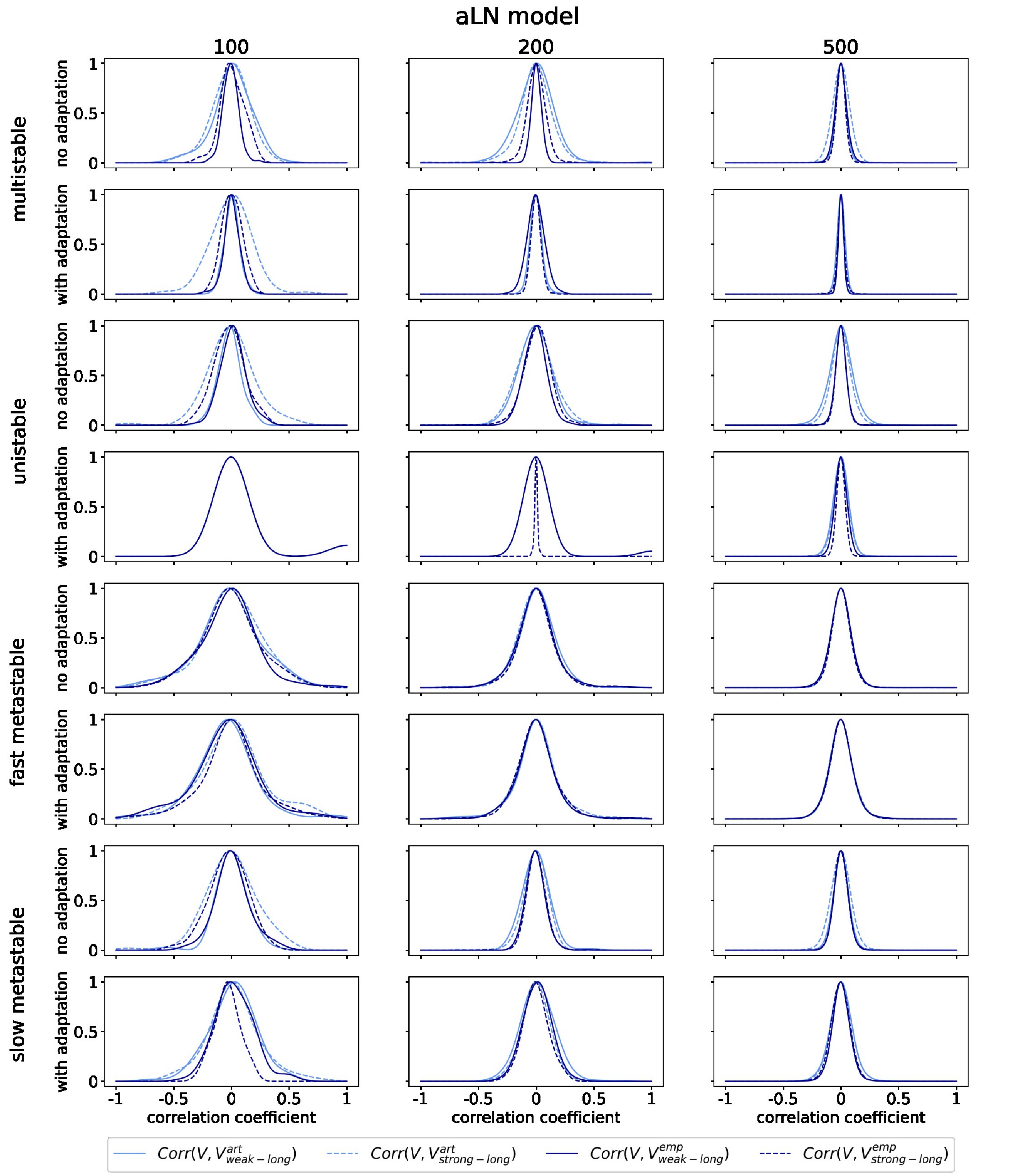

Figure A16: Distribution of the values from the matrices $Corr(V, V^{type}_{strengh})$ of correlation coefficients, each normalized to its maximum value. Correlation coefficients are computed between the spatial modes obtained with the averaged connectivity matrix C and with the spatial modes of the empirically derived (darker colors, V^{emp}) and the artificially manipulated (lighter colors, V^{art}) matrices, with stronger (dashed, $V_{strong-long}$) and weaker (solid, $V_{weak-long}$) long-range connections. Distributions are estimated using kernel density estimation. Each column corresponds to one parcellation, each pair of rows (upper row without, lower row with adaptation) to the type of stability (multistable, unistable, fast, and slow metastable). Means and standard deviations are provided in Table 3. For parameters, see A13.

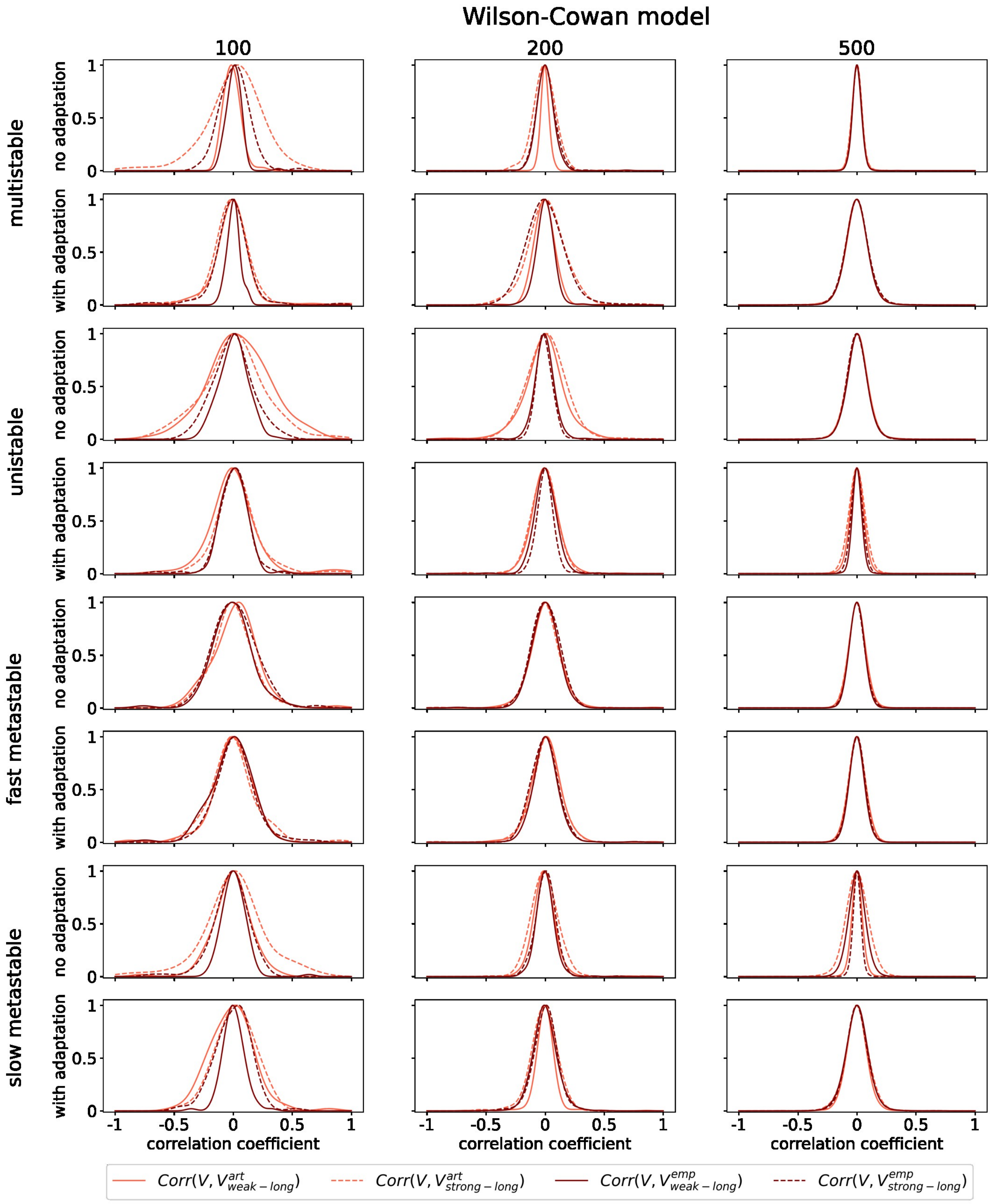

Figure A17: Distribution of the values from the matrices $Corr(V, V^{type}_{strengh})$ of correlation coefficients, each normalized to its maximum value. Correlation coefficients are computed between the spatial modes obtained with the averaged connectivity matrix C and with the spatial modes of the empirically derived (darker colors, V^{emp}) and the artificially manipulated (lighter colors, V^{art}) matrices, with stronger (dashed, $V_{strong-long}$) and weaker (solid, $V_{weak-long}$) long-range connections. Distributions are estimated using kernel density estimation. Each column corresponds to one parcellation, each pair of rows (upper row without, lower row with adaptation) to the type of stability (multistable, unistable, fast, and slow metastable). Means and standard deviatons are provided in Table A3. For parameters, see A13.

Table A3. Average standard deviation (σ) and mean (μ) of density estimates of Figure A17 for the Wilson-Cowan model, per type of stability, with and without adaptation, and per resolution. Density estimates of broadest width per resolution are highlighted with and without adaptation in **bold**. Averages over standard deviation given in bottom row.

		σ			μ		
	Resolution	100	200	500	100	200	500
Stability							
multistable	no adaptation	0.019	0.005	0.001	0.01	0.006	0
	adaptation	0.021	**0.015**	**0.008**	-0.015	0.002	0.001
unistable	no adaptation	**0.041**	**0.017**	**0.007**	-0.003	-0.006	-0.001
	adaptation	0.026	0.009	0.003	0	0.001	0
fast metastable	no adaptation	0.03	0.013	0.004	0.016	0.002	0.001
	adaptation	**0.029**	0.013	0.004	0.017	-0.003	-0.001
slow metastable	no adaptation	0.03	0.008	0.004	-0.026	0.005	0
	adaptation	0.023	0.01	0.008	-0.002	0.002	0
averages		0.027375	0.01125	0.004875			

Table A4. Overview of the parameter values used for the whole-brain aLN sleep model with 100, 200, and 500 nodes. All other parameters are given in Table 1.

Parameter	Value			Description
	100	200	500	
μ_E^{ext}	3.3 mV/ms	3.3 mV/ms	3.3 mV/ms	Mean external input to E
μ_I^{ext}	3.7 mV/ms	3.7 mV/ms	3.7 mV/ms	Mean external input to I
σ_{ou}	0.37 mV/$ms^{3/2}$	0.37 mV/$ms^{3/2}$	0.37 mV/$ms^{3/2}$	Noise strength
b	3.2 pA	3.2 pA	4.2 pA	Adaptation strength
τ_A	4765 ms	4765 ms	4965 ms	Adaptation time constant

Table A5. Overview of the parameter values used for the whole-brain Wilson-Cowan sleep model with 100, 200, and 500 nodes. All other parameters are given in Table 2.

Parameter	Value			Description
	100	200	500	
μ_E^{ext}	5.26	5.26	5.26	Mean external input to E
μ_I^{ext}	5.51	5.61	5.61	Mean external input to I
σ_{ou}	0.49	0.49	0.49	Noise strength
K_{gl}	2.18	2.18	2.18	Coupling strength
b	21.45	27	59	Adaptation strength
τ_A	1629.46	2600	2920	Adaptation time constant
v_{gl}	20	20	20	Global signal speed

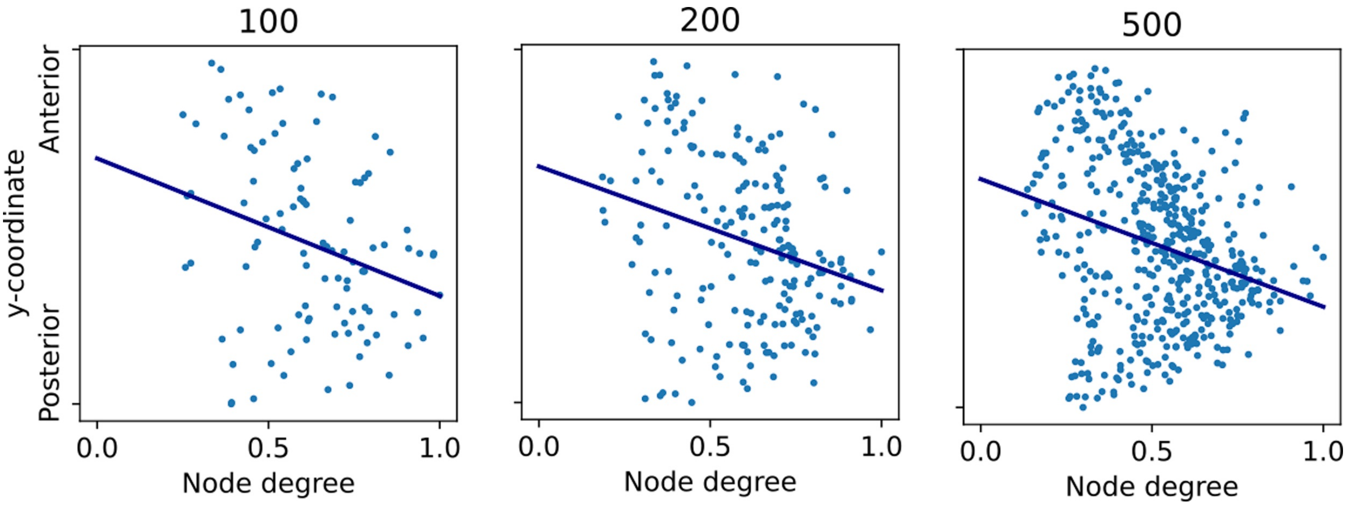

Figure A18: Correlation between node degree and the y-coordinate along the antero-posterior axis for the Schaefer parcellation scheme with 100 (left; y-slope = -62.29, r = -0.29, $p = 0.003$), 200 (middle; y-slope = -58.65, r = -0.27, $p < 0.001$), and 500 nodes (right; y-slope = -63.86, r = -0.28, $p < 0.001$).

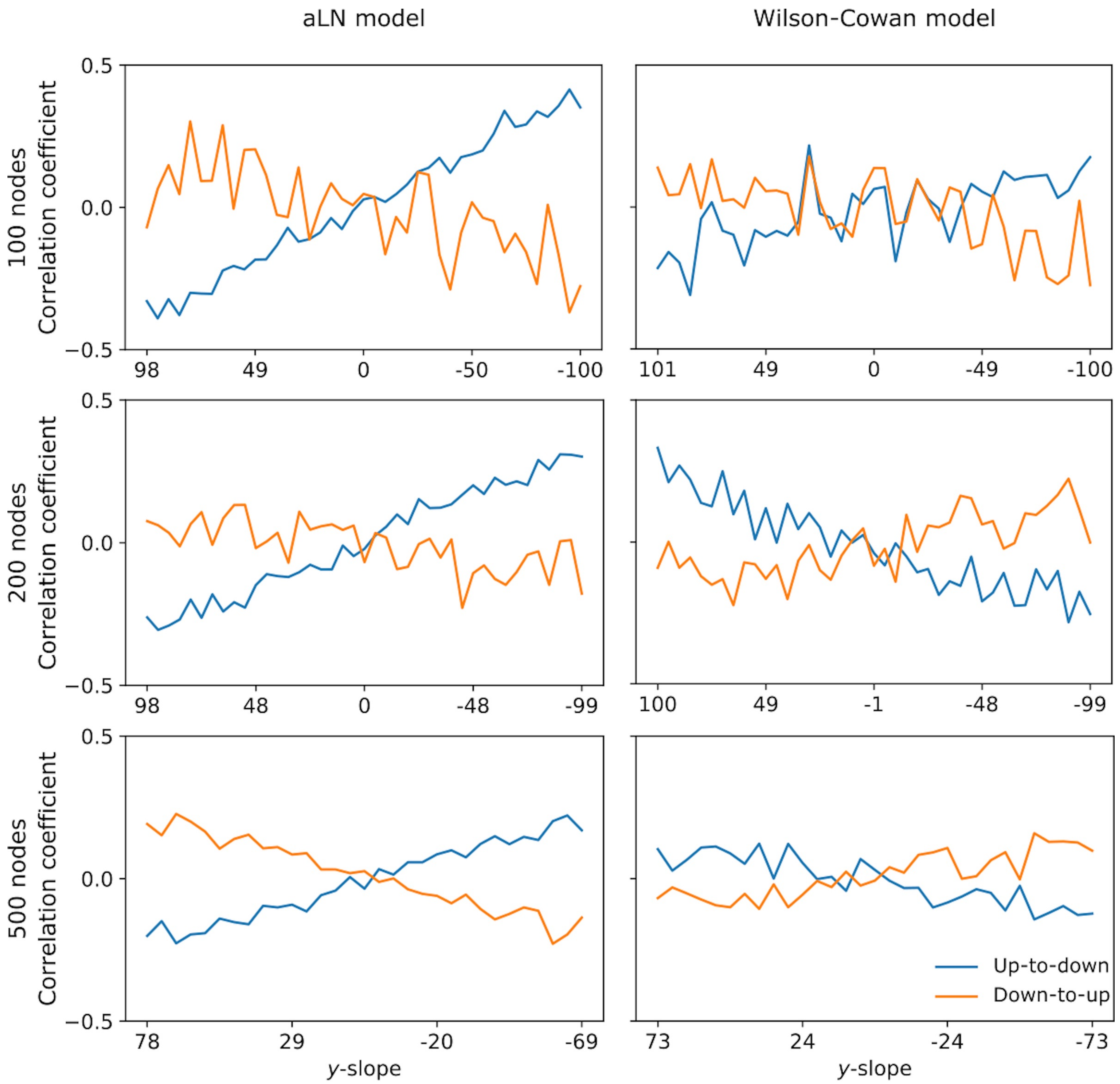

Figure A19: Correlation coefficient between mean transition phases of the nodes from the up to the down state (blue) and vice-versa (orange) and the node coordinates along the antero-posterior y-axis as a function of the structural connectivity gradient (y-slope) along the antero-posterior axis. These values were set as the targets during the permutation of the structural connectivity matrix for the aLN (left column) and the Wilson-Cowan models (right column) with 100 (top row), 200 (middle row), and 500 nodes (bottom row). The range of slope values that could be achieved through permutation was lower compared to those in Figure 10 and was additionally restricted for the 500 nodes case. Model parameters are given in Tables A4 and A5.

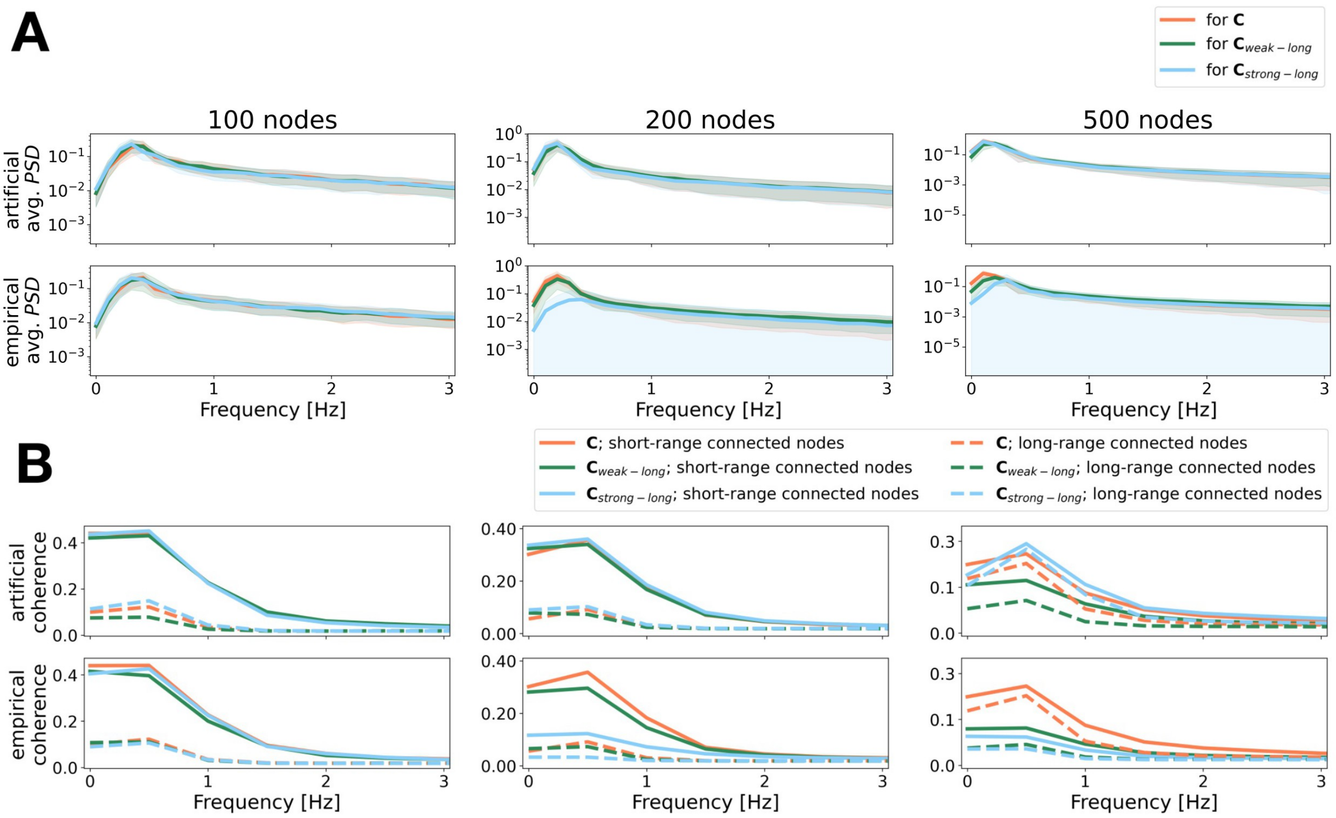

Figure A20: Power and coherence as a function of frequency for SO activity generated by the Wilson-Cowan model. Results are shown for the average connectivity matrix, C, (coral), and the connectivity matrices with weaker, $C_{weak-long}$, (green) and stronger, $C_{strong-long}$, (blue) long-range connections. Every column corresponds to one parcellation. (A) Averaged power spectra on a logarithmic scale with standard deviation for each activity induced by the three connectivity matrices. The top (bottom) row shows the results for the artificially changed (empirically selected) connections. (B) Corresponding coherence values plotted separately for nodes that are connected through short- (solid lines) or long-range (dashed lines) connections. Model parameters are given in Table A5.

Table A6. Values of the dominant temporal frequency $f_{dom} = \text{argmax}_f avg(PSD(f))$ of the averaged power spectrum and the corresponding peak power spectrum $P(f_{dom})$ of Figure 11A per parcellation for the aLN model. Values in **bold** indicate dominant frequencies f_{dom} different from 0.4 Hz which appears in most settings. Lowest row displays the standard deviation in feature per column.

		f_{dom}			$PSD(f_{dom})$		
	Resolution	100	200	500	100	200	500
artificial	$\mathbf{C}$	0.4	0.4	0.4	422.57	233.90	188.83
	$\mathbf{C}_{weak-long}$	0.4	0.4	**0.5**	404.39	296.59	224.61
	$\mathbf{C}_{strong-long}$	0.4	0.4	0.4	498.04	246.58	209.31
empirical	$\mathbf{C}$	0.4	0.4	0.4	422.57	233.90	188.83
	$\mathbf{C}_{weak-long}$	0.4	0.4	**0.5**	558.58	284.15	179.36
	$\mathbf{C}_{strong-long}$	0.4	0.4	**0.6**	482.22	124.25	97.85
Standard Deviation		0.0	0.0	0.08	59	61	44

Table A7. Values of the dominant temporal frequency $f_{dom} = \text{argmax}_f avg\big(PSD(f)\big)$ of the averaged power spectrum and the corresponding peak power spectrum $P(f_{dom})$ of Figure A20A per parcellation for the Wilson-Cowan model. Values in **bold** indicate dominant frequencies f_{dom} different from most of the other dominant frequencies per parcellation. Lowest row displays the standard deviation in feature per column.

	Property	f_{dom}			$PSD(f_{dom})$		
	Resolution	100	200	500	100	200	500
artificial	$\mathbf{C}$	**0.4**	0.2	0.1	0.21	0.44	0.75
	$\mathbf{C}_{weak-long}$	0.3	0.2	**0.2**	0.21	0.40	0.54
	$\mathbf{C}_{strong-long}$	0.3	0.2	0.1	0.23	0.46	0.73
empirical	$\mathbf{C}$	**0.4**	0.2	0.1	0.21	0.44	0.75
	$\mathbf{C}_{weak-long}$	**0.4**	0.2	**0.2**	0.19	0.34	0.41
	$\mathbf{C}_{strong-long}$	0.3	**0.4**	**0.3**	0.20	0.06	0.27
Standard Deviation		0.05	0.08	0.08	0.01	0.15	0.2

Table A8. Maximum coherence values for non-zero frequencies for the metastable states of the Wilson-Cowan model for all settings shown in Figure A20B. Both for the artificial and the empirical case, values in **bold** indicate the highest coherence values per parcellation, per set of nodes connected on a short-range (long-range). The corresponding frequencies were 0.5 Hz for all settings. Parameters are as for Figure A20.

	Property	$coh(f_{max})$					
	Resolution	100		200		500	
	Distance	short	long	short	long	short	long
artificial	$\mathbf{C}$	0.44	0.12	**0.36**	0.09	0.26	0.23
	$\mathbf{C}_{weak-long}$	0.43	0.08	0.34	0.07	0.17	0.11
	$\mathbf{C}_{strong-long}$	**0.45**	**0.15**	**0.36**	**0.10**	**0.29**	**0.27**
empirical	$\mathbf{C}$	**0.44**	**0.12**	**0.36**	**0.09**	**0.26**	**0.23**
	$\mathbf{C}_{weak-long}$	0.40	0.11	0.30	0.07	0.12	0.07
	$\mathbf{C}_{strong-long}$	0.43	0.11	0.12	0.03	0.09	0.05
